# Sex differences in cognitive performance persist into your 80s

**DOI:** 10.1007/s11357-025-01585-x

**Published:** 2025-03-17

**Authors:** Ross Julian, Stephanie Fröhlich, Katrin Müller, Melanie Dammhahn, Claudia Voelcker-Rehage

**Affiliations:** 1 Department of Neuromotor Behavior and Exercise, Institute of Sport and Exercise Sciences, University of Münster, Wilhelm-Schickard-Straße 8, 48149 Münster, Germany; 2 School of Sport and Exercise, Exercise and Sport Research Centre, University of Gloucestershire, Gloucestershire, UK; 3 Institute of Human Movement Science and Health, Faculty of Behavioural and Social Sciences, Chemnitz University of Technology, Chemnitz, Germany; 4 Institute for Neurobiology and Behavioural Biology, Behavioural Biology, University of Münster, Münster, Germany; 5 Joint Institute for Individualisation in a Changing Environment (JICE), University of Münster and Bielefeld University, Münster and Bielefeld, Germany

**Keywords:** Cognition, Older adults, Executive function, Hormones, Memory, Estrogen, Sex differences, Sex dimorphism, Testosterone

## Abstract

**Background:**

Sex differences in cognitive performance have been extensively documented. Understanding the underlying factors contributing to sex differences in older adults is imperative to promote healthy cognitive aging. Sex hormones, estrogens, and testosterone have been suggested to be associated with cognition. Nevertheless, there is a scarcity of studies investigating the sex difference in cognitive performance and the contribution of gonadal hormones in older adults. Hence, the current study aimed to investigate sex differences in cognitive performance and elucidate the association between gonadal hormones and cognitive performance in 80+ -year-olds.

**Methods:**

Using confirmatory factor analysis in a sample of 131 older adults (aged 80 to 92 years), 17 cognitive performance measures were divided into two cognitive components: executive functioning and memory. Subsequently, mediation analyses were conducted to determine the direct effect of sex and the indirect effect mediated by gonadal hormones on executive functioning and memory.

**Results:**

Females outperformed males in executive functioning and memory. However, gonadal hormones did not mediate the sex effect on cognitive performance. Estrogen levels significantly predicted executive functioning but not memory. Testosterone levels did neither predict executive functioning nor memory.

**Conclusion:**

Our study confirms enduring sex differences in memory and executive function, even among individuals aged 80 and above. Current gonadal hormone levels do not mediate these differences. While estrogen may predict executive function, its influence does not explain the sex differences. These findings underscore the complex nature of cognitive disparities between sexes in older age, warranting further investigation into underlying mechanisms.

## Background

Cognitive decline is well-documented throughout the ageing process, with a notable decrease typically observed from the mid-'50 s onward [1, 2]. This decline primarily affects fluid mental abilities, including memory, executive functioning, processing speed, and reasoning [1]. While these changes are a natural aspect of aging, there is considerable variation among older adults regarding cognitive performance and its decline rate. Besides aging, various reasons may explain this heterogeneity, such as educational level, physical fitness, lifestyle, and non-modifiable factors such as sex [3–5].

Much research has been conducted to determine the differences in cognitive performance between males and females. The results are often equivocal due to the lack of sex differences, or when differences are apparent, they are often only of a small magnitude [6]. For example, executive functions are seen as an essential set of cognitive processes necessary to control and coordinate other cognitive abilities and behaviors. A recent meta-analysis suggests no sex differences in overall executive functioning, however, there are some task-specific differences in other cognitive domains [4]. This meta-analysis from Gaillard and colleagues [4], encompassing over 600 studies, unveiled a superiority in episodic memory performance among females, accompanied by a discernible difference due to the content that was to be recalled. The results indicated that females had an advantage in verbal tasks, such as naming words, sentences, and prosing nameable images and locations (g = 0.11 – 0.28). Conversely, males have been identified to score better than females on spatial tasks involving mental manipulation and visualization of three-dimensional objects, such as mental rotation and matching with effect sizes ranging from medium to large (d = 0.56 – 0.94) [3]. Moreover, a recent meta-analysis [5] has also identified that these significant male advantages remain for visual-spatial working memory measures across 180 effect sizes from persons aged 3 to 86 years (mean d = 0.16). Interestingly, mean age was identified as a significant moderator, whereby the magnitude of the sex differences in visual-spatial working memory increased with age [5].

These sex differences in cognitive performance persist across the lifespan, including in older adulthood. Munro et al. [7] conducted a study involving 957 participants aged 67 – 88 years (480 females and 477 males), identifying that older females displayed an advantage on tests assessing psychomotor speed, verbal learning, and memory tasks. Conversely, males had an advantage on tasks involving visual construction and perception. These findings tend to suggest that sex differences observed early in life continue to manifest in older adulthood, highlighting the enduring nature of these cognitive disparities [8–10]. The current literature provides extensive information regarding cognitive performance throughout the lifespan; however, there is a notable lack of specific knowledge concerning individuals aged 80 and older. For instance, the meta-analysis conducted by Gaillard and colleagues [4] focused on studies with participants up to the age of 79, while Munro et al. [7], although including individuals aged 80 and above, analyzed individuals spanning a 21-year age range, which may limit the representation of the 80 and older adult population. Consequently, enhancing our understanding of cognitive performance in adults transitioning into their 80 s is essential, particularly given the increasing number of individuals in this age group globally.

Sex differences in cognitive performance are attributed to various biological factors, including neuronal lateralization [11, 12], region-specific brain dimorphisms [12–14], and gonadal hormone levels [15–19]. Research indicates that high circulating gonadal hormone levels correlate with observable effects on cognition in young adults, such as improved performance in visuospatial tasks with higher testosterone levels [19, 20]. Whilst estrogen has been associated with greater verbal and nonverbal memory [21], with cognitive performance varying across different age groups corresponding to estrogen concentrations [21]. Throughout life, individuals are exposed to varying levels and concentrations of hormones due to sex differentiation. Notably, cognitive decline often coincides with significant hormonal transitions such as menopause in females (average age 51), marked by rapid decreases in estrogen [18], and declines in testosterone levels in males [22]. However, these findings have only been observed across a broad age spectrum, with limited investigation focusing on much older adults, specifically those aged 80 and above. The existing literature overlooks the specific population of older adults, particularly those aged 80 and above, but also primarily relies on correlational analyses to examine the rate of change between variables. Moving beyond correlative analyses to more rigorous statistical methods could yield deeper insights into the underlying processes driving these relationships. While current observations suggest a significant role of hormones in cognitive performance, there remains a critical gap in understanding whether these hormonal influences persist within the aging population. Therefore, a more comprehensive statistical approach is necessary to elucidate the complex interactions between hormones and cognitive performance in older adults.

Sex and gonadal hormones may influence cognitive abilities, maintenance, and decline with age. However, limited research has explored the interplay between sex, hormones, and cognitive performance, specifically in 80-plus healthy older adults. Consequently, this study aimed to retrospectively investigate the associations between sex, hormonal differences, and cognitive performance within older adults of 80-plus years. By elucidating these intricate relationships, this study aimed to provide valuable insights into the underlying mechanisms contributing to cognitive performance in older adults. Our primary hypothesis posited that sex is the predominant predictor of cognitive performance differences, with females exhibiting superior performance in the cognitive assessments. Moreover, we hypothesized that estrogen is a mediating factor to explain observed sex differences in cognitive abilities, regardless of the loss of estrogen during the menopausal period.

## Methods

### Subjects

A total of 244 participants, aged 79—93 years (M = 82.5, SD = 2.5) and born between 1926 and 1939, were recruited from the Sensor-based Systems for Early Detection of Dementia (SENDA) project [23]. Table 1 includes detailed information about the recruitment process, exclusion, and inclusion criteria of the SENDA sample. Among the SENDA participants, 160 participants volunteered to provide saliva samples. However, 10 did not return their samples, 17 were cognitively impaired according to the MoCA cut-off criteria (MoCA < 23, [24], and for 2 all cognitive test scores were missing, resulting in a final sample of 131 older adults (60 males and 71 females, Table 2). Recruitment strategies and inclusion and exclusion criteria can be found in the published study protocol [23]. None of the individuals in the final sample received hormone replacement therapy at the time of the study, and no information was available about it prior to the study period. Participants provided written informed consent before participating in the study, which the Research Ethics Committee approved at the Faculty of Behavioral and Social Sciences at Chemnitz University of Technology, Germany (V-232–17-KM-SENDA-07112017). The trial was retrospectively registered at the German Clinical Trials Register (DRKS) with registration number DRKS00013167.

**Table 1:** Detailed description of the recruitment process, exclusion and inclusion criteria of the study sample

Recruitment strategies
- Calls for participation via (free) local newspapers
- Calls for participation via the official university website
- Invitation letters sent to 3300 Chemnitz residents in cooperation with the city registration office (random selection from addresses with the following criteria: German citizens, age 80 to 90 years, no nursing homes)
- Word of mouth from already enrolled volunteers
Inclusion criteria
- Age ≥ 80 years
- Independent means of travel to and from the testing facility
- German fluency at native language level
Exclusion criteria
- Medical ban from sports and other strenuous activities
- Diagnosed psychological disorders (e.g., major depressive episode, anxiety disorder, substance use disorder)
- Diagnosed neurocognitive disorders (e.g., delirium, dementia due to Alzheimer’s Disease, dementia due to vascular disease)
- Montreal Cognitive Assessment < 19
- Permanent impairments due to brain surgery or stroke
- Other neurological diseases (e.g., epilepsy, Parkinson’s disease, neuropathy)
- Severe diseases of the respiratory system (e.g., COPD stage 4, severe asthma)
- Severe diseases of the cardiovascular system (e.g., cardiac arrhythmia, heart failure, arterial occlusive disease)
- Severe diseases of the musculoskeletal system (e.g., severe arthritis, orthopedic operations in the last 6 months)
- Diabetes with diagnosed neuropathy
- Substance abuse
- Current participation in other clinical trials

**Table 2:** Sample characteristics. Data is provided as mean and (standard deviation). Sex differences were tested with Student’s t-Test or Welch's unequal variances t-Test and printed in bold when the sexes differed significantly

	Male (*n* = 60)	Females (*n* = 71)	t-Test results
Age (years)	83.68 (2.46)	83.01 (2.30)	*t*(129) = 1.60*, p* = 0.113
MoCA	25.90 (1.85)	26.72 (2.04)	***t*(129) = −2.39, *p* = 0.019**
MMSE	27.85 (1.33)	28.49 (1.17)	***t*(129) = −2.95, *p* = 0.004**
CCI^1^	1.50 (1.73)	1.39 (1.88)	*t*(114) = 0.33, *p* = 0.738
Education (years)	15.47 (3.63)	12.88 (2.47)	***t*(101.06) = 4.68*, p* < 0.001**
Estrogen^2^ (pg/mL)	5.61 (3.33)	5.66 (3.19)	*t*(128) = −0.10, *p* = 0.922
Testosterone^3^ (pg/mL)	65.24 (34.06)	40.53 (29.23)	***t*(*126)* = 4.42*, p* < 0.001**

Note. MoCA = Montreal Cognitive Assessment, MMSE = Mini-Mental State Examination, CCI = Charlson Comorbidity Index

^1^Due to missing data this was based on only 54 males and 62 females

^2^Estrogen data from 1 female was removed from the analysis because of measurement error

^3^Testosterone data from 2 females and 1 male had to be removed because of measurement error

Table 1 is taken from Fröhlich et al. [25] with permission.

## Measures

For a detailed description of all measures in the SENDA project, please refer to [23]. Cognitive assessments were performed at approximately eight-month intervals, with the number of assessments ranging from one to four depending on the enrolment date of each participant. The assessment closest to the date of saliva sample collection was chosen for each participant to represent their current cognitive status in relation to their hormone levels (estrogen and testosterone). The interval between cognitive testing and saliva sampling was between 22.0 and 93.6 weeks (M = 38.9, SD = 16.0).

### Cognitive assessment

During two measurement days, each participant underwent a battery of eleven established neuropsychological tests. This included seven subtests of the Consortium to Establish a Registry for Alzheimer's Disease Neuropsychological test battery (CERAD-NP) [26]: Boston Naming Test, Word List Learning, Word List Recognition, Word List Free Recall, Figure Drawing Task, Figure Recall Task, and the Trail Making Test. Furthermore, the Digit Symbol Substitution Test [27], the Serial Sevens Test [28], the Digit Span Forward Test [27], and the Flanker Test [29] were used. From these tests, 17 separate performance scores were obtained for each participant (Table 3). All tests were paper–pencil or oral tests carried out by trained project staff. Only the Flanker Test was a computerized assessment in which participants reacted as fast as possible via keypress to the color of the center target disk (green or red) while ignoring the opposite color (red or green) of the surrounding flanker disk (a detailed description of the same task is already available here [29]). In addition, education was recorded as the number of years spent in school and higher education according to CERAD-NP recommendations [26]. For the cognitive assessments, data was missing from 21 participants across the Trail Making Test and the Flanker Test. One participant abstained from the B condition of the Trail Making Test, and twenty participants were not present on the second measurement day where the Flanker Test was conducted.

**Table 3:** Overview of all cognitive tests administered, including the test scores obtained, a brief description of each score, the cognitive function it measures, and the corresponding factor loadings (EF = executive functions, MEM = memory) from the final confirmatory factor analysis

Test	Score description	Score name	Cognitive function	Domain
Digit Symbol Substitution Test	Number of correctly filled out symbols in 90 s	DSST	Updating	EF
CERAD Neuropsycho-logical Test Battery	Number of correctly identified words in the Boston Naming Test (0–12)	-	Visual naming	-
	Number for words recalled Trial 1 of the Word List Task (0–10)	WL1	Verbal memory	MEM
	Number for words recalled Trial 2 of the Word List Task (0–10)	WL2	Verbal memory	MEM
	Number for words recalled Trial 3 of the Word List Task (0–10)	WL3	Verbal memory	MEM
	Percent of correctly identified words in the Word List Recognition Task	Recog	Verbal memory	MEM
	Percent of correctly remembered words in the Word List Recall Task	Recall	Verbal memory	MEM
	Number of s-Words produced in one minute	Fluency.S	Verbal fluency	EF
	Number of animals produced in one minute	Fluency.A	Verbal fluency	EF
	Point score of the Figure Drawing Task (0–11)		Visuo-spatial ability	-
	Point score of the Figure Recall Task (0–11)	Fig.Recall	Visual Memory	MEM
	Trail Making Test A time	TMTA	Updating	EF
	Trail Making Test B time	TMTB	Shifting	EF
Serial Sevens Test	Number of correct answers given in 15 s	-	Updating	-
Digit Span Forward Test	Number of maximally remembered digits		Updating	-
Flanker Test	Average reaction time of incongruent trials	FL.RT	Inhibition	EF
	Accuracy of incongruent trials	FL.Acc	Inhibition	EF

### Saliva hormone assessment

Saliva sample collection was conducted according to standardized guidelines. Participants were provided with saliva sample kits and instructions. On the day following receipt of the kits, participants were asked to provide unstimulated whole saliva samples upon awakening. To account for the pulsatile nature of hormone production and achieve a more precise concentration value, five saliva samples were provided over a period of one and a half hours, with approximately 20 min between each sample. Participants were instructed to refrain from consuming excessive amounts of dairy products, alcohol, and chocolate the evening before sample collection. On the day of sample collection, participants were asked to abstain from food and drink (except water), smoking, using toothpaste, and (when possible) medication intake until the sample had been collected. Any medication taken before or during the measurement time was documented and controlled to ensure no influence on hormone concentrations. Unstimulated whole saliva samples (~ 3 mL) were collected into sterile 5 mL plastic containers and sent to the research team within 48 h of collection. The samples were immediately placed in a −20 °C freezer until assayed to maintain sample integrity.

The estrogen and testosterone concentrations in saliva were determined using commercially available enzyme-linked immunosorbent assays (ELISAs) (DRG Instruments GmbH, Marburg, Germany). Estrogens commonly refer to a group of hormones. To maintain consistency with a significant portion of the literature, we will indicate our measurement of estradiol across the manuscript as estrogen. The assays were performed in singlet in accordance with the manufacturer's instructions using the BioTek ELISA Miroplatten-Wascher ELX-50 and BioTek ELISA Mikrotiterplatten-Reader EL-800 (BioTek, Vermont, USA). The intra-assay coefficients of variation for salivary estradiol and testosterone for the collected samples were 4.6% and 7.5%, respectively. For three participants, concentration values that fell outside the normal physiological range were recorded, which warranted consideration as measurement errors. The hormone values of two female subjects were excluded from the dataset due to their hormone levels deviating from the established reference ranges: one female exhibited anomalies in both estrogen and testosterone levels, while another female showed aberrations in testosterone levels. Additionally, the testosterone value of one male subject was omitted from the analyses as his testosterone levels exceeded the accepted normal reference values.

### Statistical analyses

Statistical analyses were performed in R using MVN package for assessing multivariate normality [30] and lavaan package for structural equation modelling [31]. All structural equation models were estimated using the ‘MLR’ estimator to control for non-normality of the data and using the full-information maximum likelihood option ‘ML’ to deal with missing data in the cognitive test battery and excluded hormone concentrations. First, confirmatory factor analysis (CFA) was performed to combine the cognitive test scores into the two aggregated cognitive factors, executive function and memory, following the results from [32]. Next, the effect of sex directly and mediated via hormones estrogen and testosterone on executive function and memory were estimated while controlling for education and MoCA scores. Standardized coefficients (direct paths: β, indirect effects: *ab*), model fits and R^2^ were reported for each model. Results were considered significant at alpha = 0.05.

## Results

**Fig. 1:**
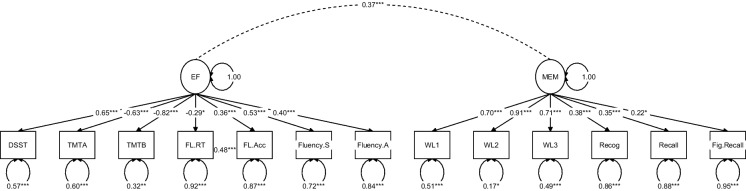
Results of the confirmatory factor analysis of the cognitive performance scores. Note. EF = Executive Functions, MEM = Memory, DSST = Digit Symbol Substitution Test, TMT = Trail Making Test, FL.RT = Flanker Test: average reaction time of the incongruent condition, FL.Acc = Flanker Test: accuracy in the incongruent condition, Fluency.S = Fluency of S-Words, Fluency.A = Fluency Animals, WWL1 = Wordlist Learning first Trial, WL = Wordlist Learning Trial 2, WL3 = Wordlist Learning Trial 3, Recog = Wordlist Recognition Test, Recall = Wordlist Recall Test, Fig.Recall = Figure Recall Test

After checking the data distribution of all cognitive scores for normality, the Boston Naming Test and the Figure Drawing Test were not included into the factor analysis because of strong ceiling effects. In addition, the Serial Sevens Test and the Digit Span Forward Test did not load significantly on the executive function factor and were removed from the CFA. The results of the CFA (Fig. 1) indicated acceptable model fit (χ^2^(63) = 94.24, *p* = 0.007; AIC = 9741.81; robust CFI = 0.93; robust RMSEA = 0.057, p (RMSEA ≤ 0.05) = 0.327, SRMR = 0.078). The two factors of the factor model were used as dependent variable in the structural equation model of the following analysis.

**Fig. 2:**
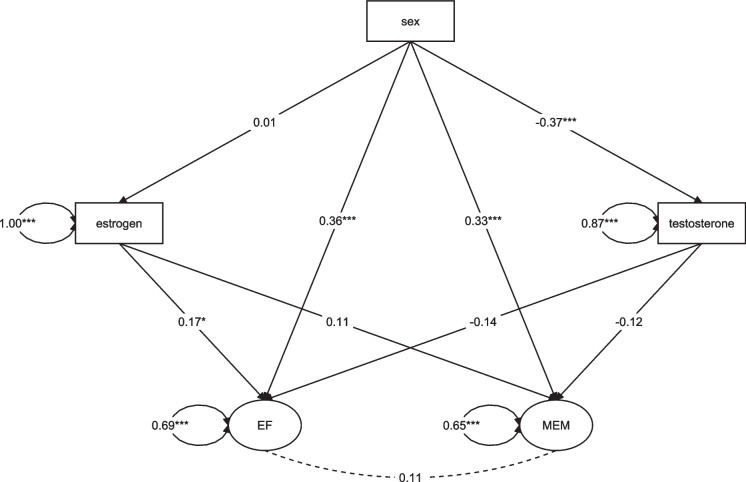
SEM results for the mediation analysis. Not depicted are the included control variables education and MoCA score as additional variables explaining the EF and MEM factor. Note. All coefficients are standardized. EF = factor Executive Functions, MEM = factor Memory

**Fig. 3:**
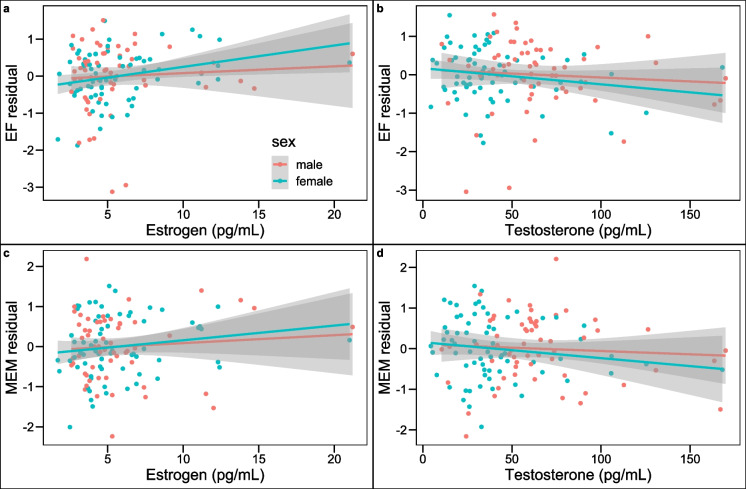
Residual scatterplot showing the relationship between **a**) executive function and estrogen, **b**) executive function and testosterone, **c**) memory performance and estrogen, and **d**) memory performance and testosterone after regressing out all other predictors. Note. EF = Executive Functions) and MEM = Memory, residual were obtained by regressing out all other predictors (sex, other sex hormone, education) of the factors in the model. Larger residuals indicate better performance

In the mediation model (χ^2^(123) = 217.35, *p* < 0.001; AIC = 11,623.24; robust CFI = 0.85; robust RMSEA = 0.071, p (RMSEA ≤ 0.05) = 0.030, SRMR = 0.082) a direct effect of sex on executive function (β = 0.36, *p* < 0.001) and memory (β = 0.33, *p* < 0.001) was revealed after controlling for education and MoCA score (Fig. 2). No significant indirect effect of sex mediated through hormones on executive function (estrogen: ab = 0.00, *p* = 0.94, testosterone: ab = 0.05, *p* = 0.25) or memory (estrogen: ab = 0.00, *p* = 0.94, testosterone: ab = 0.04, *p* = 0.23) was presented. Looking at the partial paths in the model revealed that males had significantly higher testosterone levels (β = −0.37, *p* < 0.001) while there was no difference in estrogen levels (β = 0.01, *p* = 0.94) between males and females. Estrogen was a significant predictor of executive function (β = 0.17, *p* = 0.02) but not of memory performance (β = 0.11, *p* = 0.19). Testosterone was neither significantly associated with the executive function factor (β = −0.14, *p* = 0.216) nor the memory factor (β = −0.12, *p* = 0.18). Figure 3 includes scatter plots depicting these relationships between hormones and both cognitive performance factors. Education and MoCA score were included as covariates into the model and education was significantly associated with the executive function outcome (β = 0.24, *p* = 0.01) but not with the memory factor (β = 0.07, *p* = 0.42). The MoCA score was significantly associated with both cognitive factors (EF: β = 0.27, *p* = 0.01, MEM: β = 0.37, *p* < 0.001).The included predictors (sex, estrogen, testosterone, education, and MoCA score) explained 35% of the variance of executive function and 31% of memory.

## Discussion

The study’s primary objective was to investigate sex differences in cognitive performance and explore the potential association with current levels of gonadal hormones (estrogen and testosterone). Therefore, we utilized a factor analysis to identify cognitive domains based on the applied cognitive battery developed within the SENDA project [23]. The analysis yielded two distinct domains, namely executive function and memory. Consistent with prior research, we found sex differences in memory [3, 31], specifically, females exhibited a better performance compared to males. Furthermore, we found that females outperform males in executive function, a finding that does not consistently align with existing literature [4]. Although notable sex differences were observed in cognitive performance, no indirect effect of sex mediated through hormones was detected. Albeit estrogen emerged as a significant predictor of executive function. The analysis indicated that the observed differences between sexes for memory and executive function could only be partially attributed to the included variables (Table 3). These findings imply that estrogen may exert an influence on the development of these cognitive performance metrics. Nevertheless, it is apparent that multiple additional factors likely contribute to a greater extent in explaining these differences in 80 + year-olds.

Sex differences in cognitive performance vary from small to large effects, as documented in previous studies [6]. The Gender Similarities Hypothesis posits that males and females exhibit considerable similarity across most cognitive variables, though not all. In our investigation, we identified significant differences between sexes on two cognitive domains: executive function and memory. While literature often reports no differences in executive function between sexes [4], recent meta-analyses have highlighted specific executive function tasks that show sex disparities [4]. In our CFA, verbal fluency and inhibition (Table 3) were included and contributed to the executive function factor. Across studies, females consistently demonstrated better performance in verbal fluency compared to males [32], whilst Gaillard and colleagues [4] identify that response inhibition has emerged as an executive function domain where females exhibit moderately better performance than males. Despite literature suggesting overall similarity in EF between sexes, our findings support Gaillard and colleagues' [4] suggestion that sex differences are task-dependent. Specifically, tasks within the SENDA battery that contribute to the executive function domain align with those previously documented to favor female performance over males.

Our female participants' memory performance was greater than that of their male counterparts, consistent with previous findings [3, 33]. This pattern also appears to be content-specific, indicating that the nature of the information to be remembered significantly influences recall ability. In previous studies, females have demonstrated particular proficiency in verbal memory tasks [3], while males perform better in spatial memory (e.g., remembering a route). Within our cognitive battery, the memory domain comprises six tests, of which five are verbal memory tasks (see Table 3). The observed sex differences in memory may be further accentuated by the specific tasks within this domain, contributing to the notable disparities between males and females.

It is well-established that cognition generally declines with age [1, 2]. However, research on sex differences in older populations, particularly those aged 80 and above, remains limited. Nonetheless, our findings are consistent with prior studies, indicating that healthy older adult females consistently demonstrate superior performance in tasks associated with executive function and memory across the lifespan, including advanced old age. For instance, de Frias et al. [34] examined episodic and semantic memory, and visuospatial ability in individuals aged 35 to 80 at baseline and found enduring sex differences over a ten-year follow-up period: Females exhibited superior performance in tasks assessing verbal episodic memory and verbal fluency, while males outperformed females in tasks involving visuospatial functions [34]. Additionally, studies by Maitland et al. [35], Pauls et al. [36], and Jockwitz et al. [10] further support the consistency of sex differences, particularly in the verbal versus spatial domains, among older individuals. While this study did not directly assess visuospatial domains, the derivation of the executive function domain through factor analysis, incorporating processing speed, inhibition, attentional control, and working memory (Table 3), provides a different perspective on multiple incorporated cognitive metrics. The current findings confirm that differences in cognitive abilities persist even during the later decades of life.

An additional aim of this study was to explore the influence of gonadal hormones on cognitive performance. It has long been suggested that endogenous sex hormones can impact cognitive abilities; for instance, endogenous estrogen has been linked to memory performance [37]. Our current findings revealed that levels of hormones estrogen and testosterone did not mediate the sex-related differences in executive function or memory. This observation may be attributed to the age group of the participants. As previously mentioned, certain cognitive tasks exhibit sex differences that persist throughout the lifespan. Notably, these differences are less pronounced in early and older adulthood compared to other life stages, as demonstrated in tasks involving verbal abilities [3]. Therefore, discrepancies in cognitive performance would be more evident during periods characterized by substantial differences in hormonal levels between males and females, such as adolescence and adulthood. However, the present study female cohort exclusively comprised postmenopausal females who were not undergoing hormonal replacement therapy. Consequently, the concentration levels of estrogen did not significantly differ between males and females, which may explain the lack of mediating effects.

While no mediating effects of hormones on cognitive performance were observed, estrogen emerged as a predictor of executive function performance. Estradiol, a form of estrogen, has consistently been implicated in influencing cognitive performance, as supported by numerous studies [17, 21, 38–41]. Previous research has highlighted an association between a longer reproductive window, indicative of increased exposure to estrogens, and better cognitive health in later life [42, 43]. In cellular and animal models, estrogens had neuroprotective properties, stimulating the production of brain-derived neurotrophic factors and enhancing synaptic spine density in the hippocampus [44]. These findings suggest that exposure to estrogens may contribute to the preservation of neural health in late life, potentially explaining why estrogen was found to predict executive function in our population. However, concerning the direct effects on sex differences, our study suggests that factors beyond gonadal hormonal concentrations play a role in the persistence of these differences in cognitive abilities, underscoring the multifaceted nature of sex disparities in cognition.

Sex differences in cognitive performance may also be influenced by cohort disparities, which could stem from various biological factors such as health conditions or lifestyle choices related to psychosocial gender roles, as well as systematic differences in life course experiences. Regarding biological factors, previous research has indicated that some of the cognitive advantages observed in older females may be mediated by fewer cardiovascular risk factors, including factors such as smoking, waist-to-hip ratio, and cholesterol levels compared to males [45]. It cannot be disregarded that the sex differences uncovered in our analysis may be associated with systematic differences in health between males and females. According to the Charlson Comorbidity Index [46], there were no significant differences in comorbidities between males and females in our sample, suggesting comparable health conditions between the two groups. Concerning lifestyle factors, educational attainment is of particular interest as it systematically differs between sexes, especially among older birth cohorts as examined in our study [47], and is also positively correlated with cognitive performance in older adults [48, 49]. Our analysis indicated that the sex difference observed could not be explained by differences in educational level. In our sample, females, on average, had fewer years of education yet achieved equal or better cognitive performance scores compared to male participants, a pattern consistent with previous observations [50]. This suggests that rather than explaining the sex differences, educational attainment would have obscured the findings. Numerous other lifestyle factors related to psychosocial gender roles are speculated to contribute to the sex differences in cognitive performance observed in old age [41, 45, 51]. However, as our study solely focused on current gonadal hormone concentrations and did not intend to investigate other non-biological factors, the specific contribution of these non-biological lifestyle factors to sex differences in cognitive performance remains speculative. Nonetheless, it is reasonable to assume that they at least partially contribute to our findings.

In the current study, males were not found to perform better than females across the cognitive performance measures. Additionally, no associations were observed between testosterone levels and cognitive performance across executive function and memory. Androgens, including testosterone, have been linked to cognitive performance, particularly in spatial rotation and awareness tasks. Evidence suggests that prenatal androgens influence spatial performance in girls with congenital adrenal hyperplasia [52] and serve as predictors of spatial cognition in males [53]. Furthermore, in older males, lower testosterone concentrations have been associated with poorer performance on selected cognitive tests. Although a significant difference in testosterone levels between males and females was observed in our study, as depicted in Fig. 2A, this difference did not translate into sex differences benefitting males in executive function or memory performance. One potential explanation for these findings lies within the CFA results of the SENDA test battery used in our study, which did not include specific tests assessing visual-spatial functioning. While one test of visual memory (Table 3) was included in the executive function factor, research suggests that males may have an advantage in tasks requiring visual-spatial processing, such as those involving figures, shapes, and routes [5]. However, since this test was only one of six tests comprising the executive function domain, its contribution may not have been substantial enough to detect a better male performance in executive function. Consequently, our ability to discern male abilities in cognitive tasks was limited. Future research studies employing a similar design would benefit from exploring the relationship between cognitive tasks and testosterone, particularly tasks involving high demands of visual-spatial processing.

The present study exhibits both strengths and limitations. Notably, the utilization of confirmatory factor analysis enabled the development of comprehensive variables that capture a holistic perspective of cognition, thereby reflecting real-life cognitive processes rather than isolating specific aspects.

Across research, the age range of participants often varies widely, resulting in significant diversity in cognitive and hormonal states within the population [7, 10]. By exclusively enrolling older adults aged ≥ 80 years, the current study achieved a more homogeneous sample in terms of cognitive and hormonal characteristics compared to other studies. When investigating sex differences, it is crucial to consider the various factors that might contribute to their development.

Aside from hormonal influences, genetic and social factors may also contribute to the observed sex differences. Our study extended its investigation to explore potential hormonal effects on cognitive performance while controlling for other known factors that may affect sex differences, such as education level. However, participants' general health is also a significant contributor to cognitive performance. Although the Charlson Comorbidity Index was utilized, it may not provide a comprehensive assessment of participants' health, especially considering that females in this age group may generally exhibit better overall health.

While significant differences in cognitive performance favoring females were identified in our study, there may be limitations regarding the cognitive outcomes. We choose a two-factor model with one latent factor for executive functions and the other latent factor operationalizing memory based on findings from [54]. Reliable cognitive tests were used to derive the indicators of these latent factors. Surprisingly, the Serial Sevens Test and the Digit Span Forward test did not load highly on the executive function factor and were, therefore, removed from the analysis. Because of this, it may be speculated that, rather than assessing purely executive function as intended, the factor instead or additionally represent processing speed. Disentangling the relationship between processing speed and executive function is challenging, and it has been reported that a portion of age-related deficits in executive function performance may be attributed to a slowing in information processing [55]. In our model three of the executive function indicators (Digit Symbol Substitution Test, Trail Making Test A and B) are also frequently used as measures for processing speed [56, 57]. Hence, our executive function factor may not represent a pure measure of executive function but also processing speed, it remains a useful outcome for detecting sex differences in neurocognitive aging. In particular, the use of latent factors to study sex differences is superior to other commonly used methods, such as using z-scores to obtain composite scores, which is done without checking model fit [54], or analyzing individual cognitive tests, which does not provide a comprehensive model of cognition [58].

Furthermore, it is worth noting that the absence of specific cognitive tests tailored specifically for males (e.g., spatial visual tasks) may limit the identification of tests demonstrating better male performance. Consequently, the cognitive profiles of both sexes may not have been fully represented in the study.

It is also important to recognize that these findings may not adequately reflect the broader population of individuals aged 80 and older. The study involves a relatively small sample size of 131 older adults, further reduced to two sex-specific subgroups. Furthermore, the participants were volunteers, which limited population representation due to self-selection bias, as volunteers may have distinct characteristics such as better health, greater motivation, and demographic similarities that do not reflect the broader, more diverse population. However, since participant names and addresses were primarily sourced from the records of the residents' registration office, it is likely that we reached a subset of individuals who may not have been accessible through newspaper recruitment methods. As noted in the introduction, there is a paucity of research focused on individuals aged 80 years and older, particularly regarding the role of sex hormones. This study further contributes to the current discourse and addresses existing gaps in the research landscape.

Finally, hormonal concentrations were collected retrospectively and at the same time point for all participants. However, discrepancies might exist between the time elapsed since their last cognitive assessments and their hormonal evaluations. However, this potential limitation was minimized by the study's recruitment strategy, which targeted a significant age group where substantial hormone fluctuations are not expected within the observed timeframes.

## Conclusion

Collectively, our study makes a significant contribution to the existing literature on sex differences in cognitive performance. The results robustly demonstrate the presence of sex differences in memory and task-specific executive function, with females consistently outperforming males. Notably, these differences persist into advanced adulthood, including individuals aged 80 years and above. Furthermore, our findings suggest that current gonadal hormone concentrations do not mediate these sex differences in older age groups. It is noteworthy that while estrogen predicts executive function performance, indicating its potential importance for cognitive development, this association may not extend into older age. These findings underscore the complexity of factors contributing to sex differences in cognitive abilities, warranting further research to unravel the underlying mechanisms, particularly among older adults, even at the advanced age of 80 years and beyond. Future investigations can thus focus on elucidating the interplay of various biological, environmental, and social factors in shaping cognitive performance disparities between sexes in older age groups.

## Data Availability

The datasets used and/or analysed during the current study are available from the corresponding author on reasonable request.
